# Expression and clinical significance of miR-204 in patients with hypertensive disorder complicating pregnancy

**DOI:** 10.1186/s12884-022-04501-9

**Published:** 2022-03-07

**Authors:** Xin He, Dan-Ni Ding

**Affiliations:** Department of Obstetrics, Hunan Provincial People’s Hospital, The First-Affiliated Hospital of Hunan Normal University), Jiefang Xi Lu, Changsha, 410005 Hunan China

**Keywords:** Hypertensive disorder complicating pregnancy, MiR-204, Diagnostic value, Inflammatory indexes, Receiver operating characteristic curve, Logistic regression, Person coefficient

## Abstract

**Objective:**

Hypertensive disorder complicating pregnancy (HDCP) is a unique and common obstetrical complication in pregnancy. The current study sought to investigate the diagnostic value of serum miR-204 in HDCP patients.

**Methods:**

A total of 196 HDCP patients were enrolled, with 54 healthy pregnant women as controls. The expression levels of miR-204 and inflammatory factors in the serum were determined. Receiver operating characteristic (ROC) curve was used to assess the diagnostic value of miR-204 in HDCP patients. Person coefficient was introduced to analyze the correlation between miR-204 and inflammatory indexes. Kaplan–Meier method was employed to analyze the effect of miR-204 expression on the incidence of adverse pregnancy outcomes. Logistic regression was adopted to assess the risk factors for adverse pregnancy outcomes.

**Results:**

miR-204 expression was upregulated in the serum of HDCP patients. The serum miR-204 level > 1.432 could assist the diagnosis of HDCP. miR-204 level in the serum was positively correlated with TNF-α, IL-6, and hs-CRP concentrations in HDCP patients. The risk of adverse outcomes was higher in pregnant women with high miR-204 expression. High miR-204 expression was associated with an increased risk of adverse pregnancy outcomes after adjusting the family history of HDCP, systolic pressure, diastolic pressure, AST, ALT, LDH, 24-h urinary protein, TNF-α, IL-6, and hs-CRP.

**Conclusion:**

The high expression of miR-204 assists the diagnosis of HDCP and is an independent risk factor for adverse pregnancy outcomes in HDCP patients.

**Supplementary Information:**

The online version contains supplementary material available at 10.1186/s12884-022-04501-9.

## Introduction

Hypertensive disorder complicating pregnancy (HDCP) is usually defined as the pregnant woman with blood pressure higher than 140/90 mmHg on two separate measurements with a 4-h interval at least, and with normal blood pressure before the 20th week of pregnancy [1]. HDCP is classified into four categories by the American College of Obstetricians and Gynecologists (ACOG): (1) gestational hypertension, (2) preeclampsia, (3) chronic hypertension, and (4) chronic hypertension with superimposed preeclampsia [2]. HDCP accounts for 5% to 8% in all pregnancy [3]. HDCP remains one of the leading causes of mortality and morbidity in pregnant women and infants, contributing to approximately 30,000 maternal and 500,000 perinatal deaths [4]. Panaitescu et al. investigated the incidence of HDCP in the maternity hospital in Romania and found that the incidence of preeclampsia was 1.2%, gestational hypertension was 2.2% and chronic hypertension was 0.4%, and among these, HDCP pregnancies had higher rates of stillbirth and cesarean section [5]. Inherently, HDCP is associated with insulin resistance and glucose tolerance, and diabetes mellitus type 2 increases the risk of HDCP for threefold to fourfold [6]. Moreover, the major mechanisms resulting in deaths from HDCP include intracerebral hemorrhage and cerebral edema, and lowering blood pressure timely is important to avoid the deaths [7]. Patients with HDCP have a twofold higher risk of developing cardiovascular diseases than pregnant women with normal blood pressure [6]. Therefore, early diagnosis may provide medical evidence for judging the condition and preventing adverse pregnancy outcomes to reduce maternal and fetal mortality.

microRNAs (miRNAs), a type of short RNAs with around 22 nucleotides, play a crucial role in the control of biological processes under physiological and pathological conditions [8, 9]. It is noteworthy that miRNAs are essential key players in various physiological and pathological processes during preeclampsia and gestational hypertension [10]. Low expression of miR-204-5p has been unveiled to enhance human choriocarcinoma cell proliferation and reduce apoptosis, which is probably a vital biomarker for the diagnosis, prevention, and treatment of HDCP [11]. miR-204 represses the invasion of trophoblast-like cells by targeting matrix metalloproteinase-9 [12]. Preeclampsia serum enhances caveolin-1 expression and cell permeability of human glomerular endothelial cells by downregulating miR-199a-5p, miR-199b-5p, and miR-204 [13]. Metformin treatment can prevent preeclampsia via inhibition of trophoblast cell migration by regulating the UCA1/miR-204/MMP-9 pathway[14]. Moreover, miR-204 is overexpressed in preeclampsia, and miR-204 may be conducive to the development of preeclampsia by impeding trophoblastic invasion, which could be regarded as a novel therapeutic target for preeclampsia [12, 15]. However, there are few reports about the diagnostic value of miR-204 on the HDCP. Further investigation is warranted to analyze the association between miR-204 expression and the diagnosis and prognosis in HDCP patients. Based on the aforementioned data and findings, this study aims to investigate the value of miR-204 expression on the diagnosis and adverse pregnancy outcomes in HDCP patients.

## Methods

### Ethics statement

The study was approved by the Academic Ethics Committee of Hunan Provincial People’s Hospital (The first-affiliated hospital of Hunan normal university) (Approval number: 2018–49), and all participants in this study were fully informed of the purpose of the study and had signed the informed consent before sampling.

### Study subjects

A total of 196 pregnant women in late singleton pregnancy with HDCP who were hospitalized in Hunan Provincial People’s Hospital from April 2018 to April 2021 were prospectively enrolled as the HDCP group in this study. Moreover, another 54 healthy pregnant women with singleton pregnancy were selected as the control group during the same period. The whole blood samples were collected from all participants. After centrifugation at 1500 × g for 10 min at 4ºC, the samples were stored in a freezer at -70ºC until subsequent analyses. The diagnosis of HDCP was conducted following the national guidelines (2015) and new definition.

### Inclusion and exclusion criteria

Diagnostic criteria of HDCP were as follows: normal blood pressure before pregnancy and elevated blood pressure (values of 140/90 mmHg or higher) after 20 weeks of pregnancy.

Normal pregnant women were those without immune diseases, hypertension, diabetes, and cardiovascular diseases, but with complete general clinical data; and pregnant women in late singleton pregnancy without other pregnancy complications.

Exclusion criteria were as follows: withdrew from the experiment halfway; pregnancy less than 28 weeks; complicated with hemorrhage disease, malignant tumors, severe organ dysfunction syndrome or immune diseases; no hypertension, chronic hypertension, or pregnancy-induced hypertension; loss of follow-up.

### Data collection and follow-up

The data of the study population were recorded at enrollment, including maternal age, pre-pregnancy body mass index (BMI), gravidity, and parity. The blood samples were collected from the participants to detect the levels of platelet count (PLT), aspartate aminotransferase (AST), alanine aminotransferase (ALT), and lactate dehydrogenase (LDH), systolic and diastolic pressure, fasting blood glucose, tumor necrosis factor-α (TNF-α), interleukin-6 (IL-6), high-sensitivity C-reactive protein (hs-CRP), and 24-h urinary protein. Platelet counting dilution was purchased from BioRoYee (DA0156, Beijing, China,). The fasting blood glucose of patients in the two groups was measured using an automatic biochemical analyzer. The levels of LDH, AST, ALT, TNF-α, IL-6, and hs-CRP in the serum were measured with the help of enzyme-linked immunosorbent assay (ELISA) in strict accordance with the instructions of ELISA detection kits Shanghai YSRIBIO Industrial Co., Ltd. (Shanghai, China, YS-ELISA3977, YS-ELISA2287, YS-ELISA4939, YS-ELISA1826, YS-ELISA3732, YS-ELISA3634). The protein content in urine samples was measured using urine protein test kits (KLJC0235, Shanghai Kang Lang Biological Technology Co., Ltd., Shanghai, China).

The follow-up of participants was conducted until maternal delivery and the information of maternal and fetal outcomes after maternal delivery was documented. The gestational age and pregnancy outcome were recorded. The adverse pregnancy outcomes were defined as: low birth weight, birth asphyxia, small for gestational age, premature delivery, admission of the newborn to the neonatal intensive care unit, and perinatal death. Low birth weight referred to the infants with birth weight less than 2500 g. Birth asphyxia referred to the infants with wheezing, very irregular respiration, or without respiration. Small for gestational age was defined as the birth weight of the newborn below the 10th percentile of weight distribution at the specified gestational age. Low Apgar score referred to the newborns with an Apgar score below 7 of 1-min after birth.

### Reverse transcription quantitative polymerase chain reaction (RT-qPCR)

Fasting vein blood samples (5 mL) were collected from the elbow of all participants in the morning and placed into the vacuum blood collection tubes without anticoagulants. The supernatant was separated by centrifugation at 15,000 × g for 10 min at 4ºC, stored in the freezer at -70ºC, and measured within one week. The relative expression of miR-204 in the serum was detected using RT-qPCR. The total RNA in the serum was extracted with the TRIzol reagent (Simgen, Hangzhou, China, 5,301,100) and the concentration and purity of RNA were determined using a spectrophotometer (BFMUV-2000, Bigfish, Hangzhou, China). The cDNA was synthesized using the reverse transcription kit (ZY1011R, Shanghai Zeye Biotechnology Co., Ltd., Shanghai, China). U6 acted as an internal reference for miR-204, and the primer sequences were designed by Daixuan Bio (Shanghai, China). The PCR amplification was conducted with the help of real-time fluorescent quantitative PCR analyzer (Image, Beijing, China, 100,133), and the relative expression of miR-204 after normalization to the internal reference U6 was calculated based on the 2^−ΔΔCt^ method. Primer sequences are shown in Table 1.

**Table 1:** Primer sequences of real-time PCR

Gene	Forward 5’-3’	Reverse 5’-3’
*miR-204*	GCGCGCGCGCGCGCGT	AGTGCAGGGTCCGAGGTATT
U6	GCGCGTCGTGAAGCGTTC	GTGCAGGGTCCGAGGT

*miR-204* microRNA-204

### Data analysis

Statistical analysis and mapping of data were introduced using SPSS 21.0 software (IBM Corp., Armonk, NY, USA) and GraphPad Prism 6.0 software (GraphPad Software Inc., San Diego, CA, USA). Shapiro–Wilk method was adopted to test the normal distribution of data. Enumeration data were expressed as the number of cases and percentage (n/%). Chi-square test was employed for the comparisons among groups. Measurement data were expressed as mean ± standard deviation. Independent sample *t* test was introduced to compare measurement data among groups. Receiver operating characteristic (ROC) curve was used to assess the value of miR-204 in the diagnosis of HDCP. The correlations between miR-204, and TNF-α, IL-6, and hs-CRP levels were analyzed by Person coefficient. The effect of miR-204 expression on the incidence of adverse pregnancy outcome was analyzed by Chi-square test and Kaplan–Meier method, and Log-rank method was employed for testing the difference between groups of Kaplan–Meier curves. Logistic regression was used to assess the influencing factors of adverse pregnancy outcomes. The *p* < 0.05 was regarded statistical significance.

## Results

### Clinical baseline characteristics of participants

No statistically significant differences were evident in the mean age, pre-pregnancy BMI, gravidity, parity, fasting blood glucose, and PLT between HDCP patients and normal pregnant women included in this study (*p* > 0.05). Compared with normal pregnant women, HDCP patients exhibited elevated systolic pressure, diastolic pressure, AST, ALT, LDH levels, and 24-h urinary protein, as well as increased levels of TNF-α, IL-6, and hs-CRP (*p* < 0.05) (Table 2).

**Table 2:** General baseline data comparison [n(%), mean ± SD]

Parameters	HDCP (*n* = 196)	Control (*n* = 54)	χ^2^/t	*P*
Mean age (years)	33.45 ± 3.07	32.74 ± 3.12	1.5	0.135
Pre-pregnancy BMI (kg/m^2^)	22.51 ± 2.03	22.14 ± 1.97	1.193	0.234
Gravidity (times)	2.31 ± 0.28	2.25 ± 0.24	1.436	0.152
Parity (times)	2.01 ± 0.19	1.98 ± 0.14	1.082	0.281
Systolic pressure (mmHg)	157.79 ± 13.05	114.63 ± 10.08	22.51	< 0.001
Diastolic pressure (mmHg))	101.34 ± 8.76	73.26 ± 7.14	21.65	< 0.001
Fasting blood glucose (mmol/L)	4.75 ± 0.23	4.68 ± 0.49	1.494	0.136
ALT (U/L)	19.12 ± 1.58	12.16 ± 1.61	28.55	< 0.001
AST (U/L)	32.27 ± 3.15	22.04 ± 2.19	22.4	< 0.001
LDH (U/L)	188.24 ± 12.75	153.19 ± 11.08	18.37	< 0.001
PLT (× 10^9^/L)	208.81 ± 20.57	204.16 ± 20.03	1.479	0.14
24-h urinary protein (mg/L)	249.72 ± 10.36	109.64 ± 10.05	88.54	< 0.001
TNF-α (pg/mL)	20.82 ± 2.16	8.75 ± 0.91	40.05	< 0.001
IL-6 (pg/mL)	156.05 ± 15.28	94.61 ± 9.12	28.17	< 0.001
hs-CRP (mg/L)	6.46 ± 0.43	4.53 ± 0.27	31.3	< 0.001
Family history of HDCP		-	-	-
Yes	79 (40.31)	-	-	-
No	117 (59.69)	-	-	-
Disease type (n/%)		-	-	-
Mild preeclampsia	76 (38.78)	-	-	-
Severe preeclampsia	62 (31.63)	-	-	-
HDCP	58 (29.59)	-	-	-

n(%), number of cases and percentage; mean ± SD, mean ± standard deviation; *HDCP* Hypertensive disorder complicating pregnancy, *BMI* Body mass index, *ALT* Alanine aminotransferase, *AST* Aspartate aminotransferase, *LDH* Lactate dehydrogenase, *PLT* Platelet count, *TNF-α* Tumor necrosis factor-α, *IL-6* Interleukin-6, *hs-CRP* High-sensitivity C-reactive protein

### miR-204 was upregulated in the serum of HDCP patients and had high diagnostic values

**Fig. 1:**
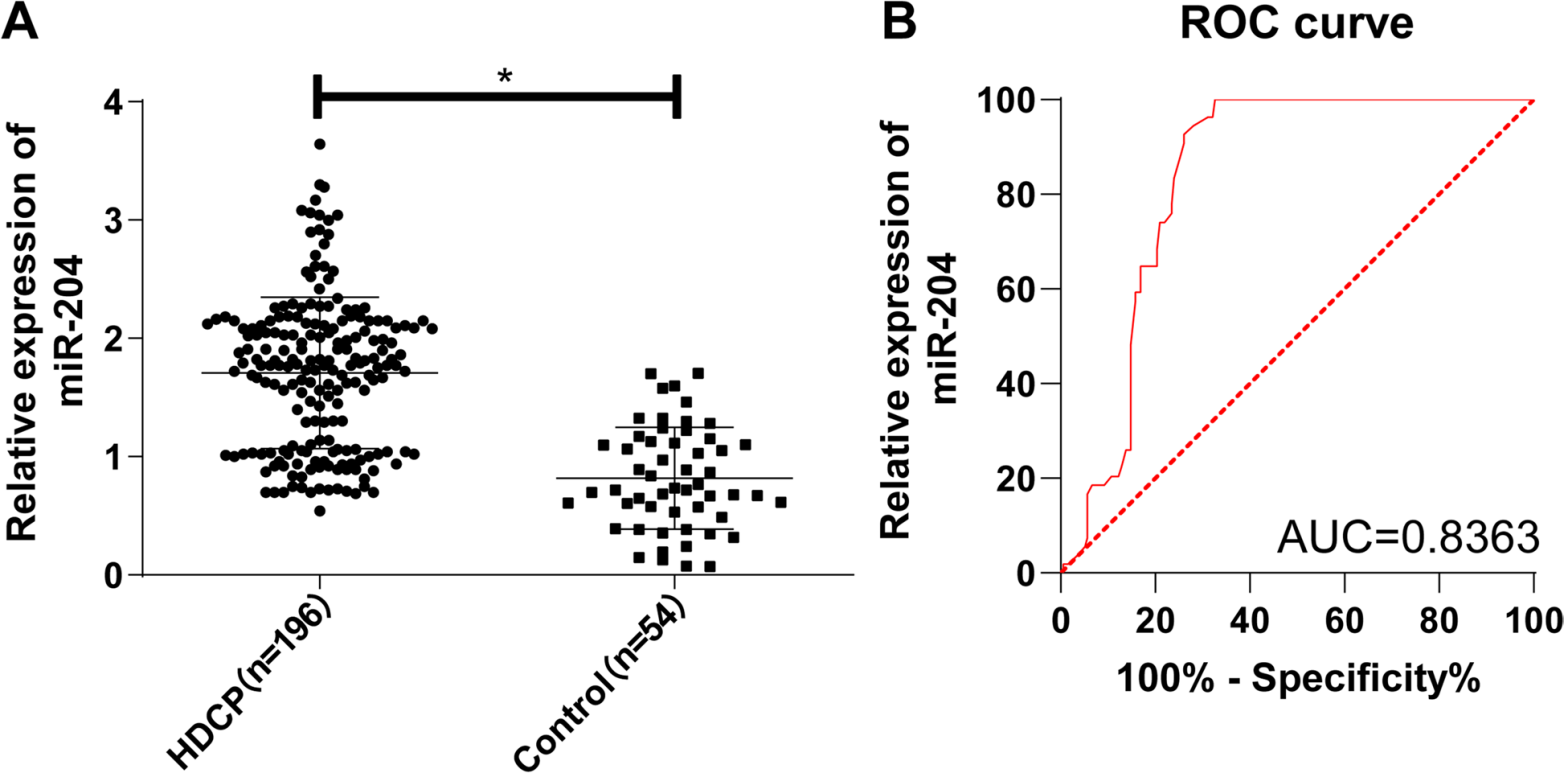
The expression of miR-204 in the serum of HDCP patients. **A** RT-qPCR was used to detect the expression of miR-204 in the serum; **B** ROC curve was employed to evaluate the diagnostic value of miR-204 on HDCP. Independent sample *t* test was used to check the data in panel A and the ROC curve was adopted to analyze the data in panel B. *p < 0.01

The expression of miR-204 in the blood between normal pregnant women and HDCP patients was compared. The results showed that the HDCP group showed a higher miR-204 expression than the control group (*p* < 0.05, Fig. 1A). Moreover, the ROC curve was plotted for the diagnosis of HDCP (Fig. 1B). The results suggested that the area under the curve (AUC) was 0.8348 and the cutoff value was 1.432 (the sensitivity was 94.44%, and the specificity was 62.24%). These results indicated that the serum miR-204 level < 1.432 could assist the diagnosis of HDCP.

### Correlation between miR-204 level in the serum and inflammatory indexes

**Fig. 2:**
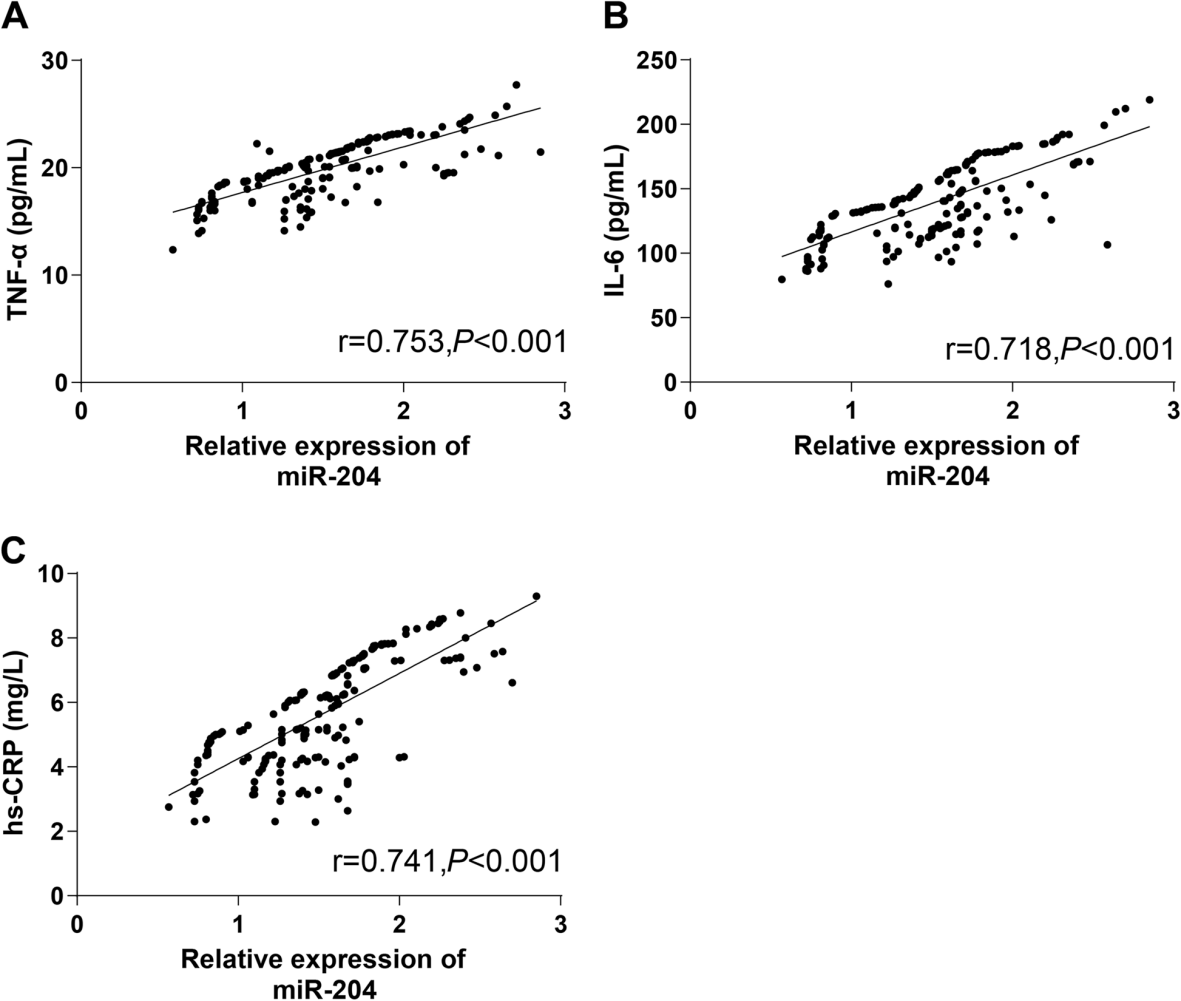
Correlation between serum miR-204 level and inflammatory indexes. **A** miR-204 level in the serum was positively correlated with TNF-α concentration in HDCP patients (*r* = 0.766, *P* < 0.001). **B** miR-204 level in the serum was positively associated with IL-6 concentration in HDCP patients (*r* = 0.692, *P* < 0.001). **C** There was a positive relevance between serum miR-204 level and hs-CRP concentration (*r* = 0.760, *P* < 0.001). Person coefficient was used to analyze the data in panel A/B/C

The secretion of inflammatory cytokines is involved in the whole development of HDCP. miR-204 is identified to play an important regulatory role in inflammatory responses [16]. To investigate the relationship between miR-204 and inflammatory responses, Person coefficient analysis was performed, which unveiled that miR-204 expression in HDCP patients was positively correlated with TNF-α, IL-6, and hs-CRP concentrations in the serum (Fig. 2A-C).

### The expression of miR-204 increased the risk of adverse pregnancy outcomes in HDCP patients

**Fig. 3:**
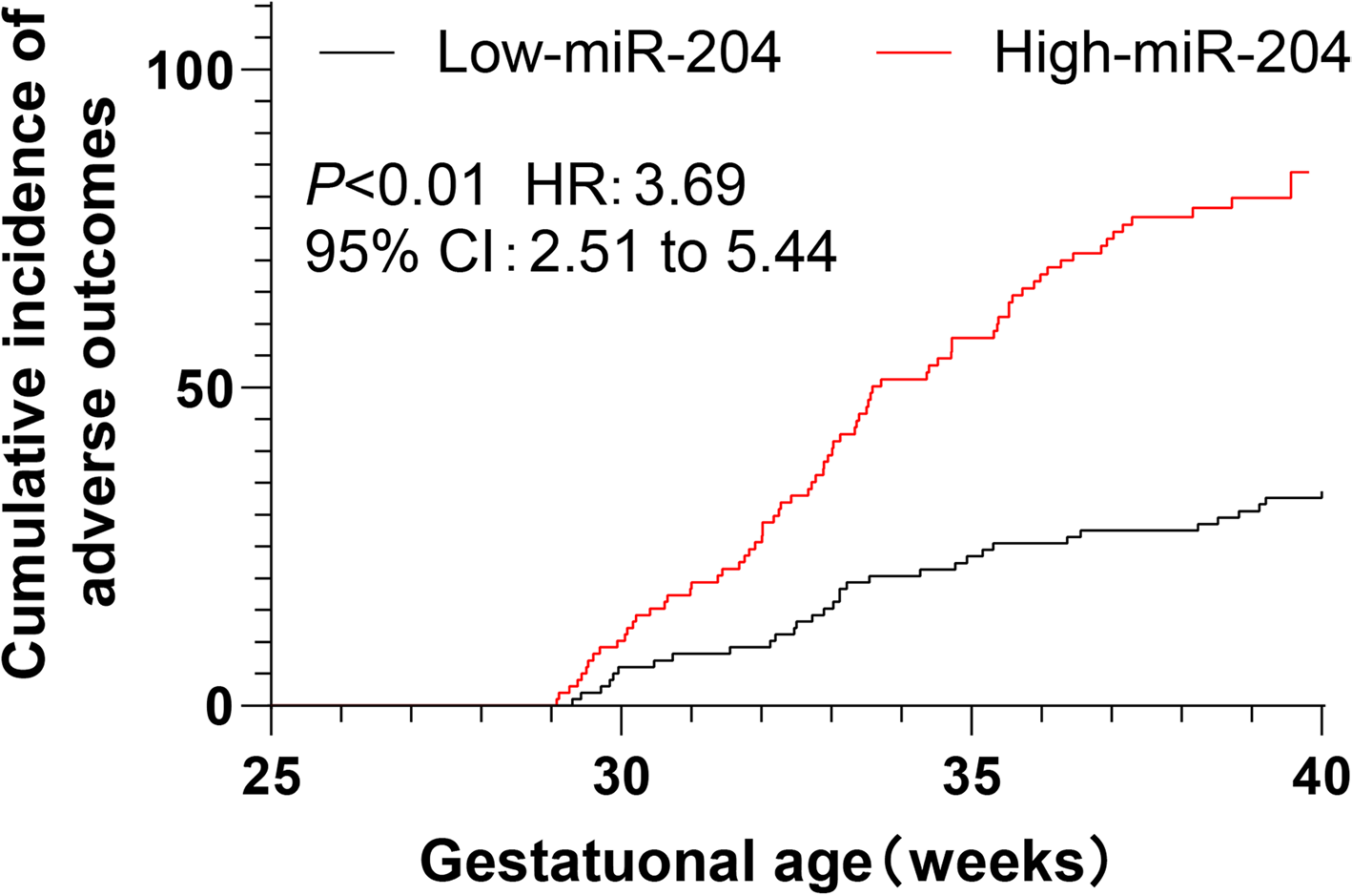
The cumulative incidence of adverse pregnancy outcomes in HDCP patients. Kaplan–Meier method was adopted to analyze the effect of miR-204 level on pregnancy outcomes in patients with HDCP. Compared with the low expression group, the curve of the high miR-204 expression group shifted left

HDCP patients were assigned into low expression group and high expression group according to the median of miR-204 expression. The incidence of adverse pregnancy outcomes between the two groups was compared, which revealed that there was a difference in the prognosis between the two groups (χ2 = 36.681, *P* < 0.001). The incidence of adverse pregnancy outcomes in the low expression group was 33.67%, which was lower than 76.53% in the high expression group (Table 3). Kaplan–Meier analysis showed that the curve of the high miR-204 expression group shifted to the left (*P* < 0.01, Fig. 3), indicating that the cumulative incidence of adverse pregnancy outcomes in the high expression group was higher in the same gestational age. Taken together, elevated miR-204 expression was associated with adverse pregnancy outcomes.

**Table 3:** Delivery status of HDCP patients with different miR-204 expression levels

	Adverse pregnancy outcome	Normal delivery	Total
Low *miR-204* expression group	33 (33.67)	65 (66.33)	98
High *miR-204* expression group	75 (76.53)	23 (23.47)	98
Total	108	88	196

*HDCP* Hypertensive disorder complicating pregnancy, *miR-204* microRNA-204

### High expression of miR-204 was independently correlated with adverse pregnancy outcomes in HDCP patients

To accurately evaluate the effect of miR-204 level on pregnancy outcomes of HDCP patients, the family history, systolic pressure, diastolic pressure, AST, ALT, LDH, 24-h urinary protein, TNF-α, IL-6, and hs-CRP (*p* < 0.05) associated with HDCP were included in Logistic multi-factor regression analysis to investigate the independent correlation between miR-204 expression and HDCP. The results indicated that after adjusting the family history of HDCP, systolic pressure, diastolic pressure, AST, ALT, LDH, 24-h urinary protein, TNF-α, IL-6, and hs-CRP levels, high miR-204 expression increased the risk of adverse pregnancy outcomes compared with low miR-204 expression (OR = 13.508, 95% CI: 1.787–102.102, all *p* < 0.05, Table 4 and 5).

**Table 4:** Logistic multi-factor regression analysis

Parameters	Variable	Valuation
Family history of HDCP	X1	No = 0, Yes = 1
Systolic pressure	X2	continuous variable
Diastolic pressure	X3	continuous variable
AST	X4	continuous variable
ALT	X5	continuous variable
LDH	X6	continuous variable
24-h urinary protein	X7	continuous variable
TNF-α	X8	continuous variable
IL-6	X9	continuous variable
hs-CRP	X10	continuous variable
* miR-204*	X11	continuous variable

*HDCP* Hypertensive disorder complicating pregnancy, *AST* Aspartate aminotransferase, *ALT* Alanine aminotransferase, *LDH* Lactate dehydrogenase, *TNF-α* Tumor necrosis factor-α, *IL-6* Interleukin-6, *hs-CRP* High-sensitivity C-reactive protein, *miR-204* microRNA-204

**Table 5:** Multi-factor analysis of influencing the adverse pregnancy outcomes on HDCP

Factors	*P*	OR	95% CI
Family history of HDCP	0.007	0.372	0.182–0.760
Systolic pressure	0.171	1.292	0.895–1.864
Diastolic pressure	0.699	1.008	0.969–1.047
AST	0.011	1.153	1.033–1.287
ALT	0.374	1.1	0.891–1.357
LDH	0.275	1.014	0.989–1.041
24-h urinary protein	0.263	0.981	0.949–1.014
TNF-α	0.549	0.894	0.621–1.288
IL-6	0.858	1.001	0.986–1.017
hs-CRP	0.634	0.933	0.701–1.241
* miR-204*	0.003	4.102	1.612–10.435

*HDCP* Hypertensive disorder complicating pregnancy, *AST* Aspartate aminotransferase, *ALT* Alanine aminotransferase, *LDH* Lactate dehydrogenase, *TNF-α* Tumor necrosis factor-α, *IL-6* Interleukin-6, *hs-CRP* High-sensitivity C-reactive protein, *miR-204* microRNA-204

## Discussion

HDCP, one of the prevailing pregnancy complications, can contribute to various adverse pregnancy outcomes and seriously harm the health of mothers and infants, yet its specific mechanism remains unclear [17]. Existing evidence suggests that miR-204 expression is elevated in patients with HDCP [11]. In this study, we illustrated the clinical significance and diagnostic value of miR-204 in the serum of HDCP patients.

First, we compared the clinical baseline characteristics of HDCP patients and normal pregnant women. The result indicated that the systolic pressure, diastolic pressure, AST, ALT, LDH, TNF-α, IL-6, hs-CRP levels, and 24-h urinary protein were increased in HDCP patients relative to those in the controls. Consistently, the hard-done works of our peers have highlighted that the changes in blood pressure, AST, TNF-α, IL-6, and hs-CRP could be employed as appropriate methods to differentiate the HDCP patients from normal individuals [18–20]. Biomarkers including ALT and LDH are considered prognostic parameters in the detection of preeclampsia severity [21]. Prior studies have documented that the expressions of TNF-α, IL-6, and hs-CRP in HDCP patients are positively correlated with the systolic blood pressure and adverse fetal outcomes of patients, which can be regarded as indexes for prognosis evaluation of HDCP patients [18]. There is also evidence to suggest that 24-h urinary protein could predict the risk of preeclampsia in pregnant patients with systemic lupus erythematosus [22]. These results could provide a reference for the clinical diagnosis of HDCP.

PNA-based microarray in placenta with severe preeclampsia demonstrated significantly overexpressed miR-204 in preeclampsia compared with the control group [15]. Therefore, we determined miR-204 expression in the serum of HDCP patients and normal pregnant women. Compared with the controls, miR-204 was upregulated in the serum of HDCP patients. Related research demonstrated that miR-204 was prominently upregulated in preeclamptic placentas [12]. Furthermore, we analyzed the diagnostic efficacy of miR-204 on HDCP using the ROC curve, which showed that the AUC was 0.8348 and the cutoff value was 1.432 with 94.44% sensitivity and 62.24% specificity, demonstrating that the serum miR-204 level < 1.432 was of great diagnostic value to HDCP. It is also noteworthy that a previous study illustrated that dysregulated miR-204 might contribute to the development of preeclampsia [12], suggesting the potential utilization of miR-204 as a diagnostic index of preeclampsia. However, there is no related research about the diagnostic value of miR-204 on HDCP. To the best of our knowledge, our study is the first to unravel the clinical value of miR-204 on HDCP, which is the main innovation of this study.

On a separate note, the levels of inflammatory cytokines in the peripheral blood of patients promote the occurrence and development of HDCP [23]. Therefore, we further detected the levels of inflammatory indexes. According to our results, miR-204 expression was positively related to levels of inflammatory indexes including TNF-α, IL-6, hs-CRP in the serum of HDCP patients. Inflammation, as a normal physiological process, has been highlighted to elevate harmful levels in preeclampsia [24]. miR-204 potentially exerts an essential effect on the regulation of inflammatory mediators and inflammation processes [25, 26]. Briefly, high expression of serum miR-204 may reflect the inflammation responses in HDCP patients.

In addition, we analyzed the relationship between miR-204 expression and adverse pregnancy outcomes in HDCP patients. The results indicated that the high miR-204 expression group possessed a higher cumulative incidence (76.53%) than the low expression group (33.67%). The curve of the high miR-204 expression group shifted to the left, indicating the elevated cumulative incidence of adverse pregnancy outcomes in the high miR-204 expression group in the same gestational age. In line with our finding, existing evidence demonstrated the involvement of the reinforced miR-204 expression in the sudden unexplained perinatal life-threatening or fatal disorders [27]. To accurately evaluate the influence of miR-204 in adverse pregnancy outcomes of HDCP patients, we further analyzed the data by Logistic multi-factor regression analysis, which illustrated that miR-204 expression was independently correlated with adverse pregnancy outcomes.

## Conclusion

The serum miR-204 expression in HDCP patients was determined for the first time in this prospective study, and the roles of miR-204 expression in the diagnosis and perinatal outcomes of HDCP were analyzed, which may provide a novel breakthrough point for judging clinical conditions and predicting adverse outcomes. However, the sample collection time in this study was from late pregnancy to before laboring, with a large time span, which may have an impact on the determination of miR-204 level. Moreover, we selected U6 as the internal control for miR-204 in RT-qPCR, but the previous study had documented that U6 may not be a widely used internal control and internal miRNA control was statistically superior to the most frequently used internal reference genes in the quantification of serum miRNAs. In the future, we will attach importance to the selection of internal control during the quantitative detection of serum miRNAs. Additionally, due to the limited conditions, we had no access to obtain the matching validation data set with a sufficient sample size to verify the RT-qPCR findings temporarily. The number of cases and events included in this study was small. Furthermore, this study failed to carry out in-depth research on the regulatory mechanism of miR-204 in HDCP cells. In future studies, we shall conduct a multicenter prospective study and expand the sample size to increase the confidence of the results obtained from the current study. In addition, the determination of serum miR-204 expression in HDCP cells could be considered to study its effect on cell proliferation, apoptosis, and cell cycle.

## Supplementary Information

**Additional file 1:** **Supplementary table 1.** Raw data of qRT-PCR.

**Additional file 2:** **Supplementary table 2.** Raw data of study subjects.

## Data Availability

All the data generated or analyzed during this study are included in this published article.

## Abbreviations

HDCP: Hypertensive disorder complicating pregnancy
ROC: Receiver operating characteristic
miRNAs: MicroRNAs
ACOG: American College of Obstetricians and Gynecologists
BMI: Body mass index
PLT: Platelet count
AST: Aspartate aminotransferase
ALT: Alanine aminotransferase
LDH: Lactate dehydrogenase
TNH-α: Tumor necrosis factor-α
IL-6: Interleukin-6
hs-CRP: High-sensitivity C-reactive protein
ELISA: Enzyme-linked immunosorbent assay
RT-qPCR: Reverse transcription quantitative polymerase chain reaction
n/%: Number of cases and percentage
AUC: Area under the curve
